# Healthy beverages may reduce the genetic risk of abdominal obesity and related metabolic comorbidities: a gene-diet interaction study in Iranian women

**DOI:** 10.1186/s13098-022-00911-z

**Published:** 2022-09-27

**Authors:** Fatemeh Gholami, Mahsa Samadi, Neda Soveid, Khadijeh Mirzaei

**Affiliations:** Department of Community Nutrition, School of Nutritional Sciences and Dietetics, Tehran University of Medical Sciences (TUMS), P.O Box 6446, 14155 Tehran, I.R. of Iran

**Keywords:** Healthy beverage index, Genetic risk score, Obesity, Metabolic markers

## Abstract

**Background & aims:**

The nutrition transition in developing countries like Iran causes the increasing rise of obesity and abdominal obesity rates. However, it is not yet well proven that environmental modifications like improving the quality of beverage intake can be effective in people who have a genetic predisposition to obesity. So, in the present study, we examine the interaction between genetic predisposition and healthy beverage index (HBI) with abdominal obesity and obesity-related metabolic risk factors in overweight and obese women.

**Method:**

Based on inclusion and exclusion criteria, 202 overweight or obese females were chosen for this cross-sectional study. Body composition, anthropometric measures, physical activity, and beverage intake data were collected and analyzed using recognized and trustworthy methodologies. Biochemical tests were performed on serum samples. A genetic risk score (GRS) was calculated based on the results of genetic tests. The predetermined HBI was calculated based on previous studies. A generalized linear model was used to estimate the interactions between GRS and HBI (GLM).

**Results:**

We found significant interactions between GRS and HBI on WHR (β = − 0.39, CI: -0.07 to 0.001, P = 0.05) and WC (β = − 6.18, CI: − 13.41 to 1.05, P = 0.09). Also, there were significant gene-diet interactions for HBI and GRS on HDL (β = 7.09, CI: − 0.73 to 14.92, P = 0.07) and FBS (β = − 9.07, CI: − 18.63 to 0.47, P = 0.06).

**Conclusions:**

These findings emphasize the HBI considering genetics appears to protect against the risks of abdominal obesity and metabolic associated obesity markers.

## Introduction

Obesity and overweight are described as excessive and abnormal fat accumulation in adipose tissue, which are linked to a variety of non-communicable diseases that result in lower quality of life and early death [1–3]. The body mass index (BMI) has limitations in terms of information on body fat content and distribution as a screening tool for overweight and obesity [1, 4].

Measures of central adiposity like waist circumference (WC) and waist-to-hip ratio (WHR) can compensate for BMI limitations and predict obesity-related disease risk [5, 6]**.** Based on previous studies, individuals with abdominal obesity had a higher chance of having elevated fasting blood sugar, high total cholesterol, high low-density lipoprotein (LDL), and low high-density lipoprotein (HDL) [6–8]. According to a population-based study in Northwestern Iran, the prevalence of abdominal obesity was estimated at 76.4%, and it was more prevalent in the female population than in men (81.4% vs. 68.6%) [9].In developing countries like Iran, due to a nutrition transition defined by shifting to high consumption of westernized diets, the overweight/obesity and abdominal obesity rates have increased [9, 10]. In western diets, beverages are the main source of excess calories, saturated fats, free sugar, and alcohol, which are related to weight gain and metabolic disease risk [11, 12]. A healthy beverage index (HBI) is a general concept that has been prepared for evaluating total beverage intake quality [13]. This dietary quality tool includes eight components of habitual beverage intake, fluid consumption, and overall beverage energy [14, 15]. Beverages intake like water, milk, fruit juice, tea, and coffee has different effects on health status [16]. Sugary beverages, such as sweetened fruit juice and sports drinks, have been linked to abdominal obesity and obesity-related conditions [15, 17]. However, the results of the studies are contradictory according to the type of drink, eating habits, and different geographical regions [18]. Sucrose sweetener is utilized in most beverages in Iran, as it is in Western countries, although the findings of western studies may not apply to other countries [19]. On the other hand, it is well acknowledged that genetic predisposition is also a significant risk factor for obesity [20]. As a result, a genetic risk score (GRS) was developed as a personalized method of avoiding or controlling obesity and associated comorbidities [21]. The genetic risk score (GRS) is calculated by adding the risk alleles for each single nucleotide polymorphism (SNP) [22]. In this context, large-scale genomic studies identified obesity-related SNPs for genes of Melanocortin-4 Receptor *(MC4R*), Cryptochrome (CRY), and caveolin (CAV) [23–25]. The identification of obesity-related SNPs led to the creation of the "gene-environment interaction" hypothesis [26]. In line with this hypothesis, compelling evidence showed that in individuals with a high intake of sugar-sweetened beverages, the genetic association between BMI and the incidence of obesity was stronger [27–29]. However, it is not yet well proven that environmental modifications like improving the quality of beverage intake can be effective in people who have a genetic predisposition to obesity. Also, most previous studies investigated the relationship between only one type of beverage consumption (usually sugar-sweetened beverage) and single SNPs, not GRS. Therefore, the present study aims to examine the interaction between BMI-GRS according to 3 SNPs such as MC4R (rs17782313), CAV-1 (rs3807992), and Cry-1 (rs2287161) with HBI on abdominal obesity and obesity-related metabolic risk factors in overweight and obese women to provide valuable insights for preventive interventions at the level of individual, family, and community lifestyles and ultimately targeted nutritional and medicinal treatments.

It is important to emphasize that our study sample is limited to overweight or obese people and excludes the general population, which includes healthy people.

## Materials and methods

### Study population

The current study is a cross-sectional study of 202 overweight or obese women aged 18 to 68, with BMIs ranging from 25 to 40 kg/m^2^. The study participants were chosen based on inclusion and exclusion criteria. Menopause, pregnancy, cardiovascular disease, diabetes, cancer, kidney disease, thyroid illness, lactation, smoking, any acute or chronic disorders, diet adherence in the previous year, weight loss supplements, and medicines to lower blood lipids and sugar are all exclusion factors. Before enrolling, all participants gave written informed consent to the ethics committee of Tehran University of Medical Sciences (TUMS) (ID IR.TUMS.MEDICINE.REC.1400.1515), which had previously authorized the study.

### Body composition and anthropometric indices

Body composition was measured by the InBody 770 scanner from InBody Co. (Seoul, Korea) based on the manufacturer's protocol. Bioelectrical impedance analysis (BIA) calculates body size measures such as weight, and body mass index (BMI). Trained research staff measured hip circumference and waist circumference, respectively, as the narrowest part of the torso and maximum posterior extension of the buttocks, with a precision of 0.5 cm. The measurement of height by using a non-stretch tape was recorded with 0.5 cm of precision. The waist-hip ratio (WHR) was calculated as the waist measurement divided by the hip measurement.

### Dietary intake assessment

Habitual dietary intake frequency over the last 12 months was assessed by using a 147-item semi-quantitative food frequency questionnaire (FFQ) whose validity and reliability have been approved previously [30]. Based on this questionnaire, the subjects were asked to report the frequency of food intake on a daily, weekly, monthly, or annual basis. The information obtained from the FFQ questionnaire was entered into an Excel program which was designed to determine the weight (grams) of each food item. Then the dietary intake was analyzed using the NUTRITIONIST 4 software (First Data Bank, San Bruno, CA) [31].

### Physical activity assessment

Each participant completed the short form of the International Physical Activity Questionnaire (IPAQ). This form consists of seven questions that divide the physical activity into four levels: intense, moderate, walking, and inactivity, and according to this criterion, the participant is placed into three categories: intense activity, moderate activity, and inactive. Since the IPAQ is a validated self-report tool, the participants were asked to report their last week activities for physical activity assessment [32].

### Healthy beverage index

For the first time, Davy and Duffey [33] developed a method to calculate Healthy beverage index (HBI). As mentioned previously, HBI is a tool for scoring the quality of overall beverage intake and categorizes consumed beverages into eight groups include water, unsweetened coffee and tea, low-fat milk (< 1.5% fat, fat-free, and/or soy milk), diet drinks (including non-calorically sweetened coffee and tea and other artificially sweetened beverages), 100% fruit juice, alcohol (including beer, wine, and liquor), full-fat milk (1.5% fat), and sugar-sweetened beverages (including fruit drinks, sweetened coffee, and tea, soda) which are shown in Table 1 [33]. Finally, total HBI points range from 0 to 100, but in the present study, the maximum score for HBI was calculated as 90 since the participants didn’t consume diet drinks and alcohol. A higher score of HBI indicates better adherence to healthier beverage intake guidelines.

**Table 1:** Healthy Beverage Index (HBI) score components

Beverage component	Description	Points
1. Water	• Water comprises at least 20% of fluid requirements • No water consumption • Water is > 0% but < 20% fluid requirements	15 0 Proportional
2. Coffee and Tea	• Unsweetened coffee and tea comprise 0%-40% fluid requirements	5
3. Low fat Milk	• < 1.5%, fat free, and /or soy milk 0%-16% of fluid requirement	5
4. Diet Drinks^a^	• Artificially sweetened beverages comprise 0%–16% of fluid requirements	5
5. 100% Fruit Juice	• 100% fruit juice comprises 0%-8% of fluid requirements	5
6. Alcohol^a^	• Between 0–1 drinks for women, 0–2 drinks for men	5
7. Full-fat Milk	• 0% of fluid requirements come from 2% fat or full-fat milk	5
8. Sugar-sweetened Beverages	• Sugar-sweetened beverages are 0%–8% of fluid requirements	15
9. Total Beverage Energy	• Energy from beverages is < 10% of total energy • Energy from beverages ≥ 15% of total energy • Energy from beverages is > 10% but < 15% of total energy	20 0 Proportional
10. Met fluid requirements	• Amount of beverages (mL) consumed was greater than or equal to fluid requirements • Amount of beverages (mL) consumed was less than fluid requirements	20 Proportional

^a^ Removed items for calculating HBI score

### Biochemical assessment

A blood sample was obtained after 10–12-h of fasting. Then all blood samples were centrifuged, aliquoted, and stored at – 80 °C for analysis by a single assay technique. Fasting blood glucose (FBS) was measured by using glucose oxidase-phenol 4-aminoantipyrine peroxidase (GOD-PAP) enzymatic endpoints, and the direct enzymatic clearance assay method was used to measure low-density-lipoprotein (LDL), high-density lipoprotein (HDL), and total cholesterol. All measurements and assessments were carried out at the Nutrition and Biochemistry Laboratory of the School of Nutritional and Dietetics at TUMS.

### Genotyping and GRS computing

Whole blood samples were collected for DNA extraction by using salting out methods [34]. For assessment of DNA integrity and concentration, we used 1% agarose gel and a nanodrop 8000 Spectrophotometer, respectively. SNP genotyping was performed by TaqMan Open Array (Life Technologies Corporation, Carlsbad, CA, USA) [35]. The forward and reverse primers of CAV-1 (rs3807992), Cry1 (rs2287161), and MC4R (rs17782313) are shown in Table 2. The fragments containing three genotypes for each gene were distinguished. Finally, we calculated GRS by summing up three single nucleotide polymorphisms (SNP) [CAV-1 (rs3807992), Cry-1 (rs2287161), and MC4R (rs17782313)] that, based on genomic associated studies like GWAS, had been linked to obesity [36–40]. According to the risk alleles for higher BMI, genotypes were coded as 0, 1, or 2 for each SNP. In this method, GRS is calculated without weighting by using the risk alleles of the three SNPs. GRS ranges from 0 to 6. Higher scores are considered as a greater genetic predisposition to higher BMI on the GRS scale [41].

**Table 2:** Information of primers

Gene Name (SNP)	Sequence	Genotypes fragment
CAV-1 (rs3807992)	Forward: 3′AGTATTGACCTGATTTGCCATG 5′	GG, GA, AA
	Revers: 5′ GTCTTCTGGAAAAAGCACATGA 3	
Cry1 (rs2287161)	Forward: 5′-GGAACAGTGATTGGCTCTATCT -3′	CC, GC, GG
	Revers: 5′-GGTCCTCGGTCTCAAGAAG-3	
MC4R (rs17782313)	Forward:5- AAGTTCTACCTACCATGTTCTTGG-3	CC, CT, TT
	Revers:5-TTCCCCCTGAAGCTTTTCTTGTCATTTTGAT-3	

### Statistical analysis

The results were analyzed by SPSS version 23.0 (SPSS, Chicago, IL, USA), and a P-value lower than 0.05 was set as statistically significant and for interaction P-value lower than 0.1 was set as statistically significant. The Hardy–Weinberg equilibrium and comparison of categorical variables were assessed with the chi-square test. The Kolmogorov–Smirnov test was used to assess the normality distribution of data. For demographic characteristics, descriptive analysis was used, and all data were reported by mean ± standard deviation. An analysis of variance (ANOVA) was applied to compare anthropometric measurements and metabolic profiles between participants. Analysis of covariance (ANCOVA) was performed to remove confounding results. To estimate the interactions between GRS and HBI, a generalized linear model (GLM) was used in both the crude and adjusted models. The adjustment was applied based on age, physical activity, energy intake, and BMI.

## Results

### Descriptive characteristics of the study sample

The current study included 202 women who were either overweight or obese. Individuals' age, weight, BMI, WC, and WHR mean and standard deviation (SD) were 36.65 ± 9.07 years, 80.75 ± 11.52 kg, 31.03 ± 3.87 kg/m2, 99.22 ± 9.60 cm, and 0.93 ± 0.05 respectively.

### The difference in means of variables across HBI

Table 3 shows the key characteristics of the study population concerning the tertile categories of the healthy beverage index. Participants in the higher tertile of HBI were older. Before adjusting for age, energy intake, and physical activity, the results displayed a significant difference across HBI for HDL. Also, participants with higher HBI had marginally significant variations in the WC (P = 0.07). After controlling for confounding variables, no significant differences across HBI were seen.

**Table 3:** Mean and SD of anthropometric measurements and metabolic factors according to tertile categories of healthy beverage index (HBI)

Variables†	HBI				
	T1(n = 69)	T2(n = 62)	T3(n = 71)	P-value	P-value _b_
Age (years)	34.02 ± 8.96	36.54 ± 8.83	38.59 ± 7.33	**0.006**	**0.001**
Body weight (Kg)	81.68 ± 12.80	80.84 ± 11.23	76.83 ± 9.18	**0.02**	**0.02**
Anthropometric measurements					
BMI (Kg/m^2^)	31.21 ± 4.49	30.75 ± 3.55	30.01 ± 3.18	0.17	0.08
WC (cm)	99.25 ± 10.64	98.49 ± 9.10	95.76 ± 8.25	**0.07**	0.09 ^a^
WHR (ratio)	0.92 ± 0.05	0.93 ± 0.05	0.91 ± 0.04	0.32	0.55 ^a^
Metabolic factors					
FBS (mg/Dl)	87.39 ± 9.32	86.17 ± 6.69	87.05 ± 11.71	0.78	0.72
Total cholesterol (g/dl)	183.43 ± 30.57	177.01 ± 31.00	178.20 ± 34.04	0.54	0.70
TG (mg/dl)	124.188 ± 76.31	117.85 ± 58.76	118.83 ± 58.97	0.86	0.88
HDL (mg/dl)	50.15 ± 9.66	46.46 ± 9.31	45.66 ± 10.02	**0.03**	0.11
LDL (mg/dl)	99.45 ± 21.86	96.44 ± 21.05	97.71 ± 22.52	0.77	0.77

p-values are in bold

*SD* Standard deviation, *BMI* Body mass index, *WC* waist circumference, *WHR* waist height ratio, *FBS* fasting blood sugar, *TG* Triglyceride, *LDL* Low density lipoprotein, *HDL* High density lipoprotein, *CHO/HDL* total cholesterol/ high density lipoprotein

a: BMI considered collinear and this variable adjusted for age physical activity and total energy intake

b: Adjusted for age, BMI, physical activity, and total energy intake

^†^ Calculated by analysis of variance (ANOVA)

p < 0.05 was considered significant

### Difference in the means of variables across GRS

There were no significant differences in cardio-metabolic parameters across GRS (Table 4).

**Table 4:** Mean and SD of anthropometric measurements and metabolic factors according to tertile categories of GRS

Variables†	GRS				
	T1	T2	T3	P-value	P-value _b_
Age (years)	36.51 ± 8.90	36.12 ± 8.40	36.46 ± 8.36	0.96	0.85
Body weight (Kg)	79.84 ± 10.74	78.32 ± 11.42	80.52 ± 11.91	0.57	0.74
Anthropometric measurements					
BMI (Kg/m^2^)	30.08 ± 3.49	30.42 ± 3.49	31.46 ± 4.23	0.07	0.15
WC (cm)	97.16 ± 8.89	97.08 ± 9.11	99.01 ± 10.31	0.40	0.44
WHR (ratio)	0.92 ± 0.05	0.92 ± 0.04	0.93 ± 0.05	0.66	0.51
Metabolic factors					
FBS (mg/Dl)	87.26 ± 9.25	85.73 ± 6.74	87.22 ± 11.33	0.67	0.59
Total cholesterol (g/dl)	183.52 ± 31.48	180.90 ± 31.00	173.71 ± 32.73	0.21	0.20
TG (mg/dl)	119.32 ± 57.27	108.73 ± 51.49	129.33 ± 78.80	0.28	0.37
HDL (mg/dl)	48.02 ± 9.14	49.07 ± 11.16	45.30 ± 9.35	0.12	0.17
LDL (mg/dl)	100.33 ± 20.45	99.73 ± 22.63	93.61 ± 22.27	0.17	0.15

*SD* Standard deviation, *BMI* Body mass index, *WC* waist circumference, *WHR* waist height ratio, *FBS* fasting blood sugar, *TG* Triglyceride, *LDL* Low density lipoprotein, *HDL* High density lipoprotein, *CHO/HDL* total cholesterol/ high density lipoprotein

a: BMI considered as collinear and this variable adjusted for Age, physical activity, and total energy intake

b: Adjusted for age, BMI, physical activity, and total energy intake

p < 0.05 was considered significant

^†^ Calculated by analysis of variance (ANOVA)

### Dietary intake of participants according to HBI

Higher HBI was linked to more tea and coffee, fruits, refined grains, carbohydrates, potassium, calcium, and fiber consumption (Table 5).

**Table 5:** Dietary intake according to tertile categories of healthy beverage index (HBI)

Variables†	HBI			P-value
	T1(n = 69)	T2(n = 62)	T3(n = 71)	
Healthy beverage index	60.98 ± 2.79	64.95 ± 0.79	69.76 ± 2.53	< 0.001
Food group				
Whole grains (g/d)	64.51 ± 62.39	66.34 ± 53.67	61.18 ± 55.35	0.86
Fruits (g/d)	490.36 ± 285.04	486.02 ± 373.68	616.99 ± 364.35	**0.04**
Vegetables (g/d)	377.60 ± 267.85	344.07 ± 220.22	431.64 ± 185.45	0.08
Nuts (g/d)	12.02 ± 11.73	13.97 ± 13.41	16.23 ± 17.90	0.23
Legumes (g/d)	45.38 ± 44.90	49.99 ± 39.85	51.18 ± 40.78	0.69
Vegetable oils	22.18 ± 21.22	24.57 ± 19.34	22.57 ± 19.82	0.77
Tea and coffee (ml/d)	294.54 ± 210.42	569.36 ± 288.07	1120.22 ± 447.93	** < 0.001**
Fruit juices (ml/d)	17.56 ± 38.79	9.42 ± 16.97	8.38 ± 12.53	0.07
Sugar-sweetened beverages(ml/d)	27.54 ± 59.10	25.40 ± 58.24	15.48 ± 35.86	0.34
High-fat milk (ml/d)	53.11 ± 146.89	29.59 ± 60.26	15.43 ± 38.15	**0.01**
Low-fat milk (ml/d)	79.43 ± 119.05	114.78 ± 119.42	134.06 ± 160.100	0.13
Water percent	51.17 ± 33.12	56.97 ± 28.67	60.89 ± 26.02	0.14
Refined grains (g/d)	337.37 ± 154.95	333.54 ± 160.87	419.78 ± 256.15	**0.01**
Animal fat (g/d)	3.52 ± 6.12	4.44 ± 7.24	4.55 ± 7.81	0.64
Dairy (ml/d)	339.26 ± 245.19	400.59 ± 178.25	421.63 ± 238.29	0.08
Eggs (g/d)	21.06 ± 13.23	19.18 ± 13.25	22.18 ± 11.54	0.39
White meat (g/d)	52.28 ± 61.53	38.94 ± 24.58	46.01 ± 42.90	0.25
Red meat (g/d)	21.48 ± 16.86	21.26 ± 17.91	21.97 ± 19.79	0.97
Nutrient intake				
Energy (kcal/d)	2426.62 ± 694.58	2513.79 ± 665.45	2803.88 ± 738.93	**0.005**
Protein (g/d)	85.41 ± 31.79	84.46 ± 21.15	94.65 ± 28.18	0.06
Carbohydrate (g/d)	338.06 ± 98.54	350.44 ± 109.83	411.44 ± 125.41	** < 0.001**
Total fat (g/d)	89.00 ± 33.81	93.72 ± 28.56	96.64 ± 32.63	0.36
PUFA (g/d)	19.42 ± 9.51	20.47 ± 8.21	20.00 ± 7.96	0.78
SFA (mg/d)	25.75 ± 11.33	27.80 ± 9.92	29.07 ± 10.77	0.18
Sodium (mg/d)	4060.59 ± 1361.02	4199.86 ± 1361.74	4377.300 ± 1395.89	0.39
Potassium (mg/d)	3886.73 ± 1564.17	4010.96 ± 1295.67	4910.49 ± 1534.29	** < 0.001**
Calcium (mg/d)	1053.63 ± 398.42	1147.62 ± 352.22	1272.97 ± 432.03	**0.005**
Vitamin C (µmol/L)	185.49 ± 104.54	183.19 ± 166.09	204.60 ± 112.08	0.56
Total fiber (g/d)	42.60 ± 17.97	41.91 ± 16.26	49.89 ± 19.81	**0.01**

p-values are in bold

Data are mean ± SD

*HBI* healthy beverage index, *PUFA* polyunsaturated fatty acid, *SFA* Saturated Fatty Acid

P < 0.05 was considered significant

### Interaction between HBI and GRS on abdominal obesity and markers for metabolic associated obesity status

In a multivariate-adjusted model controlling for confounders, we found a significant interaction between GRS and HBI on WHR; higher (β = − 0.39, CI − 0.07 to 0.001, P = 0.05) or moderate (P = 0.07) HBI adherence was more related to lower levels of WHR among individuals with higher GRS (T3). Higher and moderate HBI were associated with lower WC (β _T3_ = − 6.18, CI: − 13.41 to 1.05, P = 0.09, β _T2_ = − 8.29, CI − 16.78 to 0.18, P = 0.05) among people with higher GRS when compared to the reference group (Table 6).

**Table 6:** The interaction between GRS and HBI on anthropometric measurements and metabolic factors

Variable	GRS	T1	T2						T3					
			Crude			Model 1			Crude			Model 1		
			B	CI	P	B	CI	P	B	CI	P	B	CI	P
Anthropometric measurements														
BMI (Kg/m^2^)	T1	Reference	Reference						Reference					
	T2		− 0.61	− 4.11 to 2.89	0.73	− 0.29	− 3.77 to 3.19	0.87	0.35	− 3.20 to 3.91	0.84	0.43	− 3.13 to 4.00	0.81
	T3		− 2.83	− 6.18 to 0.50	**0.09**	− 2.27	− 5.57 to 1.22	0.21	− 1.59	− 4.47 to 1.28	0.27	− 1.15	− 4.04 to 1.74	0.43
WC (cm)	T1	Reference	Reference						Reference					
	T2		− 2.60	− 11.30 to 6.09	0.55	− 2.47	− 11.12 to 6.18	0.57	− 0.50	− 9.32 to 8.32	0.91	− 1.59	− 10.46 to 7.28	0.72
	T3		− 8.78	− 17.10 to − 0.47	**0.03**	− 8.29	− 16.78 to 0.18	**0.05**	− 6.13	− 13.30 to 1.04	**0.09**	− 6.18	− 13.41 to 1.05	**0.09**
WHR (ratio)	T1	Reference												
	T2		− 0.01	− 0.06 to 0.03	0.46	− 0.01	− 0.06 to 0.02	0.43	− 0.01	− 0.06 to 0.03	0.56	-0.02	− 0.07 to 0.02	0.89
	T3		− 0.04	0.02 to − 0.08	**0.08**	− 0.04	− 0.08 to 0.005	**0.07**	− 0.03	− 0.07 to 0.005	**0.08**	− 0.39	− 0.07 to 0.001	**0.05**
Metabolic factors														
FBS (mg/dl)	T1	Reference	Reference						Reference					
	T2		− 7.57	− 17.11 to 1.96	0.12	− 5.96	− 14.98 to 3.06	0.19	− 11.81	− 21.64 to -1.98	**0.01**	-9.07	− 18.63 to 0.47	**0.06**
	T3		0.66	− 8.49 to 9.82	0.88	1.76	− 7.08 to 10.60	0.69	− 3.72	− 12.06 to 4.60	0.38	− 1.26	− 9.20 to 6.68	0.75
Total cholesterol (g/dl)	T1	Reference	Reference						Reference					
	T2		− 17.74	-48.86 to 13.38	0.26	− 14.56	− 43.84 to 14.72	0.33	− 0.62	− 32.69 to 31.43	0.96	− 0.45	− 31.46 to 30.55	0.97
	T3		− 21.09	− 50.99 to 8.79	0.16	− 15.06	− 43.77 to 13.63	0.30	1.85	− 25.37 to 29.02	0.89	7.04	− 18.74 to 32.82	0.59
TG (mg/dl)	T1	Reference	Reference						Reference					
	T2		− 14.76	− 80.79 to 51.25	0.66	− 2.65	−64.92 to 59.61	0.93	4.64	− 63.38 to 72.66	0.89	23.76	− 42.18 to 89.70	0.48
	T3		− 19.07	− 82.49 to 44.34	0.55	− 6.72	− 67.77 to 54.32	0.82	− 32.86	− 90.56 to 24.84	0.26	− 14.66	− 69.49 to 40.17	0.60
HDL (mg/dl)	T1	Reference	Reference						Reference					
	T2		− 3.56	− 12.60 to 5.46	0.43	− 4.19	− 13.08 to 4.69	0.35	6.14	− 3.16 to 15.45	0.19	4.12	− 5.29 to 13.53	0.39
	T3		2.02	− 6.65 to 10.70	0.64	2.23	− 6.48 to 10.94	0.25	7.99	0.10 to 15.89	**0.04**	7.09	− 0.73 to 14.92	**0.07**
LDL (mg/dl)	T1	Reference	Reference						Reference					
	T2		− 15.34	− 36.49 to 5.81	0.15	− 12.43	− 32.59 to 7.72	0.22	− 7.44	− 29.23 to 14.35	0.50	− 4.60	− 25.95 to 16.74	0.67
	T3		− 10.26	− 30.58 to 10.05	0.32	− 5.88	− 25.64 to 13.87	0.55	− 0.55	− 19.03 to 17.93	0.95	3.93	− 13.81 to 21.69	0.66

p-values are in bold

GLM was performed to identify the interaction between GRS and H-PDI on cardiometabolic risk factors

Model 1 = adjusted for potential confounding factors including (age, energy intake, physical activity, and BMI)

*T* tertile, *SD* Standard deviation, *HBII* healthy beverage index, *GRS* Genetic risk score, *BMI* Body mass index, *WC* waist circumference, *WHR* waist height ratio, *FBS* fasting blood sugar, *TG* Triglyceride, *LDL* Low density lipoprotein, *HDL* High density lipoprotein

a: BMI considered as collinear and this variable adjusted for Age, physical activity, and total energy intake

P < 0.1 was considered significant

In both the crude and adjusted models (adjusting for age, energy intake, physical activity, and BMI), there were significant gene-diet interactions for HBI and GRS on HDL (β = 7.09, CI: − 0.73 to 14.92, P = 0.07) and FBS (β = -9.07, CI: − 18.63 to 0.47, P = 0.06). Participants in the third tertile of the GRS with highest HBI following compared to those in the first tertile of HBI, had greater HDL. For other factors, no significant interactions between GRS and HBI were detected (Table 6).

## Discussion

In the present study, we investigated the interaction between HBI and BMI-GRS, including MC4R (rs17782313), CAV-1 (rs38 07992), and Cry1 (rs2287161) on abdominal obesity and markers for metabolically associated obesity status in overweight and obese women. According to our findings, a higher HBI score regarding genetic predisposition potentially elicits favorable effects and appears to be a protective factor against central obesity and obesity-related metabolic markers.

HBI is focused on eight beverage groups, total beverage energy, and fluid consumption [42]. As compared to the effect of a single beverage, HBI provides the most comprehensive estimates of overall beverage quality and hence better identification of improvements in health-related outcomes. A higher value represents better adherence to beverage guidelines and healthier beverage intake patterns [43, 44].

In comparison to lower GRS, we discovered a negative significant correlation between higher GRS and both high and moderate HBI on WC and WHR. Another novel significant inverse interaction was found between the top tertile of HBI and GRS when placed in the second tertile in comparison with the lower GRS on FBS. Being in the third compared to the first tertiles of HBI and GRS, we also found a positive significant GRS-HBI interaction on HDL. There were no significant interactions between the other variables tested. Indeed, the findings of this study shed light on a previously unknown relationship between the healthy beverage index and genetic predisposition to obesity and obesity-related metabolic risk factors. There has been no previous research on the HBI and GRS interaction, and even less has looked at the interrelationship of the HBI with a variety of markers. Sugar-sweetened beverage overconsumption is hypothesized to be involved in genetic susceptibility to obesity [45–47], and individuals with a stronger genetic predisposition to obesity may be more exposed to the adverse effects of sugar-sweetened beverages on BMI [45, 48]. The potential mechanisms accounting for the obtained interactions in our literature are increased total energy intake [49, 50] along with high amounts of rapidly absorbable carbohydrates in sugar-sweetened beverages, resulting in enhanced insulin resistance [51]. Therefore, modest evidence for the observed strong GRS-HBI interactions on the reduced trend of anthropometric indices and FBS might be attributed to a decrease in sugar-sweetened beverage consumption. On the contrary, the combined genetic effects on BMI and risk of obesity appeared to be attenuated in approximately 30% of participants consuming one or more cups of coffee than those consuming less than one cup of coffee [52]. These significant interactions between coffee consumption and genetic predisposition influencing BMI, obesity, and related markers may be associated with the antioxidant, hypoglycemic, and hypolipidemic properties of biologically active compounds in coffee [53–56]. Another explanation for this relationship depends on the possible health benefits of several BMI-associated genes in the brain and hypothalamus with essential roles in energy homeostasis, food preference, and appetite control [57]. More so, higher tea consumption displayed tendencies for elevated HDL, and findings were not modified by genetics [58]. Other HBI components such as milk have been established as the main source of saturated fats [59]. In this vein, data from a cross-sectional study demonstrated strong interactions between SFA (Saturated Fatty Acids) intake and GRS for BMI, WC, and WHR and indicated that populations with a high obesity GRS may be more SFA-sensitive [60]. As a result, in our study, a significant decrease in high-fat milk consumption and an increase in low-fat milk consumption appear to represent healthy beverage selection and a protective link against overweight/obesity and lipid abnormalities. As a result, calcium and a vitamin found in dairy products, may help to enhance lipid profiles [61], HDL-to-LDL cholesterol ratio [62], and induces weight loss [63] probably through the mediation of increased fecal fat excretion. As such, these findings are believed to underlie the prevention of abdominal obesity and metabolic-related markers and corroborate that higher HBI scores may be more suitable for individuals with elevated scores of GRS to modify the aforementioned risk factors.

According to our knowledge, there are no comparable studies that compare HBI and markers such as MC4R (rs17782313), CAV-1 (rs3807992), and Cry-1 (rs2287161) with abdominal obesity and obesity-related metabolic risk factors in overweight and obese women across time. We employed BMI-GRS rather than particular single SNPs to identify high-risk groups and predict interactions between GRS and healthy beverage index with related risk variables as a strength. However, there are some limitations to this study that should be recognized. For starters, the data were cross-sectional, ruling out any causal relationship. Additionally, the study population's sample size is relatively small. Furthermore, as the HBI scoring system is based on the American population, the guidelines for beverage consumption are partial. Another limitation is the reliance on self-reported dietary data, which is subjected to report inaccuracies. Since our study only included overweight and obese women, we were not able to extrapolate our findings to all populations. Finally, individuals with the highest HBI score may consume healthier foods, [64, 65] and people with lower HBI score (lower overall water consumption) may adhere to Western dietary pattern [66], given their abilities to influence our observed associations.

## Conclusion

This study highlights the importance of healthy beverage selection in individuals with elevated GRS to modify and attenuate genetic risks of obesity and metabolic-related comorbidities. However, as a result of the limited types of literature performed in this regard, further studies are needed to fully address this issue.

## Data Availability

The datasets used and/or analyzed during the current study are available from the Khadijeh Mirzaei on reasonable request.

## Abbreviations

BMI: Body mass index
CAV: Caveolin
CRY: Cryptochrome
GLM: Generalized linear model
GOD-PAP: Glucose oxidase-phenol 4-aminoantipyrine peroxidase
GRS: Genetic risk score
FBS: Fasting blood glucose
FFQ: Food frequency questionnaires
HBI: Healthy beverage index
HDL: High-density lipoprotein
IPAQ: International Physical Activity Questionnaire
LDL: Low-density lipoprotein
MC4R: Melanocortin-4 Receptor
SFA: Saturated fatty acids
SNP: Single nucleotide polymorphism
WC: Waist circumference
WHR: Waist-to-hip ratio
